# People as frontliners in the management of disasters and public health emergencies

**DOI:** 10.3934/publichealth.2026014

**Published:** 2026-02-27

**Authors:** Phatthranit Phattharapornjaroen, Amir Khorram-Manesh, Gülcan Taşkıran Eskici, Yuwares Sittichanbuncha, Lesley Gray

**Affiliations:** 1 School of Paramedicine, Faculty of Health Science Technology, College of Medical Science, Chulabhorn Royal Academy, Bangkok 10210, Thailand; 2 HRH Princess Chulabhorn Disaster and Emergency Medicine Center, Chulabhorn Royal Academy, Bangkok 10210, Thailand; 3 Department of Surgery. Institute of Clinical Sciences, Sahlgrenska Academy, University of Gothenburg, Gothenburg 41345, Sweden; 4 Center for Disaster Medicine, University of Gothenburg, Gothenburg 40530, Sweden; 5 Gothenburg Emergency Medicine Research Group (GEMREG), Sahlgrenska University Hospital, Gothenburg 41345, Sweden; 6 Department of Nursing Administration, Faculty of Health Sciences, Ondokuz Mayis University, Samsun 57270, Türkiye; 7 Faculty of Health Science Technology, College of Medical Science, Chulabhorn Royal Academy, Bangkok 10210, Thailand; 8 Department of Primary Health Care, Faculty of Medicine, University of Otago, Wellington 6242, New Zealand

**Keywords:** community engagement, disaster preparedness, education, simulations, surge capacity, scoping review

## Abstract

Managing healthcare effectively during disasters and public health emergencies (DPHEs) is often challenging due to inadequate surge capacity across four key areas: Staff, stuff, space, and system. Expanding this capacity is vital when healthcare facilities are threatened or infrastructure fails. A promising approach involves utilizing community resources and latent individual capabilities, especially in resource-limited settings where community participation is critical. In this study, we utilized a scoping review methodology to investigate strategies for mobilizing individual capabilities within DPHE management. A systematic search was conducted across PubMed, Scopus, Web of Science, and grey literature sources. From an initial pool of 512 records, 50 documents met the inclusion criteria following a rigorous full-text review. Collaborative thematic analysis of included studies identified seven major themes: Communities as frontline actors and the evolution of the “frontliner” concept; effectiveness of community-based training and participatory interventions; participation, agency, and community-delivered health services in displacement and recovery contexts; institutionalization and integration of community capacity; trust, social capital, and local leadership as determinants of engagement and effectiveness; participatory tools for planning and the limits of “participation” in practice; and community engagement in public health emergencies and epidemic response. Leveraging local capacities and resources in community centric DPHE management can augment formal response systems as long as they are effective, sustained, and meaningful. However, significant gaps remain regarding implementation, sustainability, power dynamics, and equity. Future research needs to move beyond descriptive accounts and focus on strategies to overcome barriers to participation, ensuring equity and fair distribution of resources and rigorously evaluating the real-world impacts of integrated community–institutional approaches. Ultimately, these strategies can foster collective action to improve community health and well-being and shift the paradigm from institutional reliance to community-centric resilience.

## 1. Introduction

Disasters and public health emergencies (DPHEs) refer to interconnected crises. A Disaster involves the overwhelming socioeconomic and physical loss that breaks down overall community function. A public health emergency (PHE) focuses on the urgent and substantial risk to life and health, demanding immediate medical and public health mobilization. While major disasters often include a PHE component (e.g., sanitation failure), a focused health crisis (like an epidemic) can be declared a PHE independently of a broader community disruption. The term DPHE was introduced in the early 2000s to highlight this connection between the two event types [1]. Because DPHEs simultaneously disrupt community functioning and generate acute health needs, they place extraordinary and often sudden demands on health systems, directly linking these events to the need for surge capacity (SC).

Efficient healthcare management of DPHEs fundamentally relies on SC, i.e., the healthcare system's ability to rapidly increase its services above baseline operations to handle an extraordinary, high-volume demand for medical care during a major event like a disaster or epidemic, and across four core elements: Staff, stuff, space, and systems (4S) [2],[3]. This capacity surge management aims to mobilize the necessary quantity of trained personnel, essential instruments and devices, suitable physical areas for care, and the established instructions and plan to act professionally [2],[4]. While the initial response relies on 4S surge capacity, prolonged or DPHEs inevitably require a sustained second-round provision of these elements. Although robust planning often incorporates this risk, the increasing scale of modern DPHEs can overwhelm standard contingency plans, necessitating novel approaches [2],[3].

There are no true “alternatives” to the concept of SC (since capacity must always be met), but there are strategic models and approaches that complement, enable, or reduce the need for SC by managing the flow and distribution of demand. These strategies often fall into two major categories: Demand reduction and system integration/load balancing. The former are strategies that reduce the number of people needing high-level care. Examples include promoting community self-care, establishing telemedicine/triage hotlines to redirect low-acuity patients, and implementing aggressive public health measures to slow transmission. Many demand-reduction strategies rely directly on community-level capabilities, behaviors, and decision-making, positioning communities as active contributors to surge mitigation rather than passive recipients of care. Less common system integration/load balancing strategies share the patient load across a region to prevent any single facility from collapsing. Examples include utilizing alternate care sites (ACS) for less-critical patients, and regional coordination to divert ambulances and transfer patients to hospitals with available staff, supplies, and space (the 3 S's). These approaches increasingly recognize communities as distributed extensions of formal health systems when appropriately engaged and coordinated. Moreover, these complimentary approaches are not alternatives and are used together under a medical surge management system, assisting in effectively handling a mass casualty event [5].

The SC approach and planning should consider new partnerships with other organizations or communities. This usually starts with healthcare-related organizations, hospitals, and prehospital units across all scales of governance [2],[6]. One important aspect of this kind of collaboration is the need for common collaborative factors, by which a partnership may start through continuous and effective communication from simple coordination of resources to cooperatively performing a mission and assessing its outcomes, leading to a collaborative partnership with mutual targets and goals [7]–[9]. This is necessary to develop adequate mitigation and protective strategies. However, such partnerships are increasingly understood to extend beyond formal institutions to include communities as operational partners within DPHE management systems.

Another critical aspect often overlooked in many management plans is the integration of public resources, knowledge, and capabilities into the overall resource pool [10],[11]. Elements frequently missing include public facilities, logistical support, and, most importantly, community members as active participants and stakeholders. Their involvement significantly influences all phases of disaster management; mitigation, preparedness, response, and recovery, by enhancing collaboration, resource availability, and operational effectiveness. When properly engaged, communities contribute not only to protective measures but also to education and capacity-building efforts, improving resilience and reducing the adverse impacts of incidents [10],[12],[13].

The acronym CSCATTT (command & control, safety, communication, assessment, triage, treatment, and transport) is a foundational mnemonic used in prehospital management education, such as major incident medical management and support (MIMMS), utilized globally since the 1980s. While traditionally applied within professional emergency medical services, several components of CSCATTT, particularly safety, communication, assessment, and triage, increasingly involve community members during the critical period before formal responders arrive.

Studies have shown that CSCATTT can be used to support collaboration among organizations, including high-reliability organizations and community entities in the crucial prehospital and hospital management phases of DPHEs [14]–[18]. Continuous improvement in pre-hospital and hospital care can decrease mortality and morbidity from mass casualty incidents(s) and DPHEs. However, a lack of resources may hinder such improvement by influencing emergency medical service (EMS) response time, a crucial factor for victims' survival outcomes. Although response times of 10–20 minutes are standard depending on a city's size and traffic, delays due to resource shortages or infrastructure issues are known to worsen medical outcomes [19]. Additionally, this gap leaves victims without proper timely care. For instance, terror incidents in Paris and Boston highlighted the need for better prehospital preparedness to capitalize on this window of opportunity. Immediate response from suitably trained and educated citizens could fill this gap while waiting for EMS, increasing the chances of patients receiving exceptional care at hospitals [16]–[18].

Since communities are often directly and physically affected by DPHEs, the World Health Organization (WHO) has highlighted the importance of community engagement in achieving resiliency and protection against hazards (natural, technological, and human-made emergencies [3],[18],[20]. They stress not only in treating the injured but also to prevent injuries worsening, and such engagement is necessary to change the paradigm of reactivity to DPHEs into proactivity, which mandates a new structure in DPHEs management and encourages public involvement [3],[21]. Nevertheless, community engagement in DPHE management requires new approaches and progressive work through several steps, including risk communication and hazard identification, disaster risk reduction education, data collection, and ability to respond. Initiatives such as the Hartford Consensus and CitizenAID aim to involve citizens in MCI management through step-by-step education [22]–[26]. Other studies have shown that people are willing to help manage DPHEs, and their willingness increases after receiving appropriate education [11]. People's involvement in a resource-limited setting might be crucial for the success of any health intervention or program [11],[26]. Despite widespread endorsement of community engagement, the literature remains fragmented across disciplines, hazards, and levels of action, with limited synthesis of how individual capabilities are intentionally identified, mobilized, and integrated into DPHE management systems.

In this scoping review, we aim to synthesize knowledge on strategies for harnessing individual capabilities in managing DPHEs. We provide an in-depth understanding of the landscape and offer recommendations for effective community engagement, focusing on how individual-level capabilities function as the operational foundation of community-based DPHE response. Harnessing individual capabilities is defined as the intentional process of identifying and mobilizing the diverse skills, knowledge, and resources within citizens, viewing them as active assets rather than passive recipients of aid. This strategy is operationalized through community engagement, which establishes the necessary trust and mechanisms to utilize local knowledge and expertise. This integration of individual action forms the crucial bottom-up component of a Whole-of-Society Approach, a governance framework that strategically coordinates all sectors, Government, Private Sector, Civil Society, and Individuals, to maximize collective resilience and preparedness [27],[28].

## 2. Materials and methods

Guided by established frameworks for conducting scoping reviews, such as the Preferred Reporting Items for Systematic Reviews and Meta-Analyses extension for Scoping Reviews (PRISMA-ScR) and the Joanna Briggs Institute (JBI) Manual for Evidence Synthesis [29],[30], our primary objective of this scoping review was to systematically map the breadth and nature of available evidence on methods and strategies for leveraging individuals' potential within DPHE management frameworks. Secondary objectives included identifying key concepts, examining the types of activities individuals were involved in, exploring the facilitators and barriers to individual engagement, and formulating recommendations for enhancing community participation in DPHE preparedness, response, and recovery, focusing on models of individual empowerment [31].

### 2.1. Research questions

The following research questions guide this scoping review: a) What are the individuals' potential roles and contributions in DPHE management? b) What strategies and methods have been explored or implemented in DPHEs to utilize individuals' capabilities effectively? c) What factors facilitate or hinder the engagement of individuals in DPHE management activities? d) What recommendations can be drawn from the knowledge base to enhance the involvement and utilization of individual potential in future DPHEs?

### 2.2. Eligibility criteria

#### 2.2.1. Inclusion criteria

In this review, we considered a broad range of evidence to capture the diverse nature of approaches and perspectives. We included peer-reviewed journal articles (original research, reviews, opinion pieces), reports from governmental and non-governmental organizations, guidelines, and other relevant grey literature. Furthermore, sources discussing the roles, involvement, potential, capacity, or engagement of individuals, citizens, volunteers, or community members in any phase (preparedness, response, recovery, mitigation) of disasters or public health emergencies were considered. The geographical context was not restricted: however, only sources published in English were included due to resource limitations. There were no restrictions on the publication date to capture all available historical knowledge.

#### 2.2.2. Exclusion criteria

We excluded proceedings, book and book chapters, and papers that did not discuss the keywords. In addition, sources solely focused on the roles of formal first responders, military personnel, or professional healthcare providers without discussing the involvement of the general public or community members were also excluded.

### 2.3. Search strategy

A comprehensive search strategy was developed and executed across databases and platforms to identify relevant published and grey literature.

#### 2.3.1. Databases and search terms

The search was conducted in databases relevant to health, social sciences, and disaster studies, such as PubMed, Scopus, and Web of Science. These databases were chosen since their outcomes in combination cover a broad area in medicine and social sciences.

Search terms were developed based on the key concepts of the research questions, including terms related to “disasters”, “public health emergencies”, “emergency management”, “individual potential”, “community engagement”, “participation”, “volunteers”, “capabilities”, “skills”, “knowledge”, “preparedness”, “response”, and “recovery”. Boolean operators (AND, OR) and proximity operators were used to combine terms effectively. The search terms were adapted for the specific syntax and controlled vocabulary (e.g., MeSH terms in PubMed) of each database. Several combinations were tested to finally decide on one unique combination of keywords: *(“individual potential” OR “individual capabilities” OR “community participation” OR “volunteer engagement” OR “community capabilities” OR “individual contribution”) AND (“disaster management” OR “public health emergency management” OR “emergency response” OR “disaster preparedness”)*.

#### 2.3.2. Grey literature and search terms

To capture a broader range of evidence, including reports, policies, and guidelines not typically found in peer-reviewed journals, we conducted searches using Google and Google Scholar. Targeted search strategies included:

- Searching for specific file types (e.g., .pdf).
- Restricting searches to specific organizational websites (e.g., government agencies, international organizations, NGOs).
- Combining search terms with keywords such as “report”, “guidelines”, “policy”, and “lessons learned”.
- Applying snowballing techniques by reviewing reference lists of included documents and scanning the websites of key organizations identified during the review.

Additionally, we consulted specialized repositories such as WHO IRIS and ReliefWeb to enhance the comprehensiveness of the grey literature search.

### 2.4. Source selection

Following the execution of the search strategy, identified records were imported into reference management software (Rayyan). Duplicates were removed. The selection process involved two stages:

#### 2.4.1. Title and abstract screening

Four independent reviewers screened the titles, and four independent reviewers screened the abstracts of all records against the eligibility criteria. Records deemed potentially relevant by at least one reviewer were advanced to the next stage. Any disagreement was resolved through discussion.

#### 2.4.2. Full-text review

**Figure 1.**
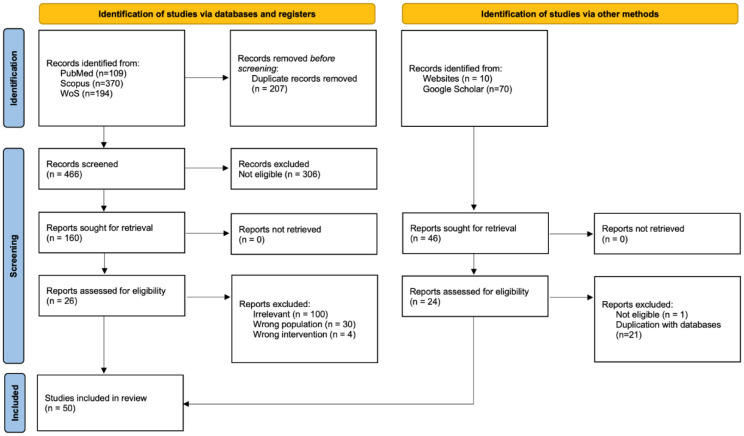
PRISMA flowchart for scoping reviews showing data identification and the selection process.

The full text of all records deemed potentially relevant during the initial screening was retrieved and reviewed in full by two independent reviewers against the eligibility criteria. Reasons for exclusion at this stage were recorded. Disagreements were resolved through discussion. A PRISMA-ScR flow diagram was used to document the source selection process (Figure 1).

### 2.5. Data extraction

A data extraction form was developed and piloted on a subset of included sources. The form captured key information from each source, including author(s) and publication year, source type (e.g., journal article, report, and guideline), geographical context of the study/report, type of DPHE addressed, specific strategies or approaches for harnessing individual potential, types of individual capabilities involved, reported benefits of individual engagement, reported challenges or barriers to individual engagement, recommendations for engaging individuals, study design or methodology (for empirical studies), key findings, and conclusions relevant to the research questions. Data extraction was performed by one reviewer and verified by a second reviewer to ensure accuracy and consistency.

### 2.6. Charting, synthesizing, and reporting the results

The extracted data was collated and presented in a charting table, providing a descriptive summary of the included sources and their characteristics. A narrative approach was used to summarize and report the findings concerning the research questions. This involved: Describing the volume and characteristics of the included sources (e.g., publication trends over time, geographical distribution, and types of publications), grouping and summarizing the different methods documented for engaging individuals in DPHEs, identifying and categorizing the various skills, knowledge, and resources that individuals contribute, synthesizing the reported advantages and obstacles of individual involvement, and gathering and presenting the recommendations provided in the literature for effective individual and community engagement.

The findings were presented thematically, guided by the research questions. The narrative synthesis provided a comprehensive overview of the knowledge landscape, highlighting key themes, variations in approaches, and areas where evidence was limited. Recommendations for engaging people in the management of DPHEs were explicitly drawn from the synthesized findings.

The thematic analysis serves as a qualitative research method for scrutinizing data, involving the exploration of a dataset to pinpoint, dissect, and communicate recurring patterns. This analysis, known as collaborative thematic analysis, can be conducted independently or collaboratively. It aims to identify pivotal themes within the data, to understand the interrelationship between themes and their manifestation in the dataset, and to utilize themes to foster fresh insights into a phenomenon [31],[32].

## 3. Results

The results obtained in the scientific database search and the grey literature are presented below. The level of evidence for each included article was appraised using the Critical Appraisal Skills Programme (CASP) checklist [33] and the Joanna Briggs Institute (JBI) critical appraisal checklist [34] (see supplementary file Table S1).

### 3.1. Source selection and characteristics

The primary search in scientific databases resulted in 673 articles (PubMed = 109; WoS = 194, and Scopus = 370). After removing 207 duplications, 466 unique database articles remained. An additional 46 documents were identified through grey literature search (including conference proceedings and reports), resulting in a total of 512 unique articles for screening. During the initial screening phase, 11 articles reached a consensus for inclusion, and 306 were consensually excluded. The remaining 195 articles with conflicting decisions (including uncertainties) were resolved through critical evaluation, which identified 39 additional articles for full-text review. This brought the total for full-text evaluation to 50 documents (Figure 1 and Table 1).

### 3.2. Overview of the evidence base

The included literature (1990–2025) comprised conceptual and practitioner-oriented papers, qualitative case studies, program descriptions, participatory methods studies, and a smaller subset of quasi-experimental/experimental evaluations. The evidence base was heterogeneous in methodological rigor and outcome measurement, reflecting the interdisciplinary and applied nature of community engagement research in DPHE contexts. Across this corpus, communities were framed not only as recipients of aid but as active agents whose capacities shape preparedness, response, and recovery trajectories [35]–[37]. The studies varied considerably in scale (individual to national), hazard type (acute disasters, epidemics, climate-related events), and governance context, contributing to thematic breadth but limiting direct comparability. Seven themes were identified across the reviewed literature, which are presented below:

#### 3.2.1. Communities as frontline actors and the evolution of the “frontliner” concept

Early work showed frontline capacity within locally trusted actors, particularly women and nursing networks, highlighting their role in planning advocacy, health education, and bridging formal and informal response systems [38]. These studies emphasized social trust and proximity as key mechanisms enabling early action in resource-constrained settings.

In parallel, neighborhood preparedness organizations were described as potential sources of triage and treatment capacity after disasters while introducing the enduring concern that uncoordinated spontaneous volunteers may increase harm without structured training and integration [39]. Later, the literature increasingly formalized this idea into a tiered model distinguishing “immediate responders” (bystanders/community members) from formal first responders, with policy emphasis on universal skills training, liability protection, and integration with incident management systems [40].

#### 3.2.2. Effectiveness of community-based training and participatory interventions

Across intervention-focused studies, community-based training was associated with improvements in preparedness knowledge, confidence, and self-efficacy. Simulation and drill-based training showed larger gains in procedural knowledge and confidence than lecture-only approaches [41]. Quasi-experimental participatory interventions demonstrated statistically significant improvements in preparedness knowledge, attitudes, and reported practices in intervention compared with control communities [42]. Targeted CERT programming for high-risk adolescents similarly produced substantial pre–post gains in preparedness knowledge and self-efficacy, alongside high stated willingness to assist others in a real event [43].

More recently, a women-led peer training model in coastal India showed marked improvements in knowledge scores at six months and gains in CPR skills relative to controls, supporting the feasibility of peer-to-peer “women training women” community frontliner pathways [44]. This study provides rare evidence of skill acquisition beyond self-reported outcomes and supports the feasibility of peer-to-peer, gender-focused frontliner models in high-risk settings.

#### 3.2.3. Participation, agency, and community-delivered health services in displacement and recovery contexts

Evidence from post-emergency and displacement settings indicated that community participation can improve service reach while restoring agency among affected populations. In Tanzania, a refugee-led health education model improved community awareness and was associated with higher health-seeking among households aware of the Health Information Team, suggesting that diffusion and visibility of peer-frontliner roles shape downstream service utilization [45].

Beyond immediate response, longitudinal recovery research illustrated that residents could shape planning decisions, build local capacity, and sustain risk reduction practices when participatory structures persist over time. However, these studies also documented constraints related to initial top-down recovery approaches, institutional inexperience, and uneven power-sharing, which limited the pace and scope of community influence [46].

#### 3.2.4. Institutionalization and integration of community capacity

A consistent theme was that community capacity is most effective when deliberately integrated into formal systems through training standards, coordination mechanisms, and role clarity. Professional volunteer integration models (e.g., Medical Reserve Corps) emphasized credentialing, liability, and organizational structures to operationalize surge capacity [47]. Broader planning-oriented frameworks argued for leveraging local resources as a “force multiplier,” positioning citizen participation and leadership as central to prevention, mitigation, and response [35],[48]. Community resilience initiatives in Los Angeles translated these principles into operational levers (e.g., leadership, communication, and partnerships), demonstrating baseline gaps among community organizations and documenting how coalitions applied participatory approaches to embed resilience activities into routine community life [49]–[51].

#### 3.2.5. Trust, social capital, and local leadership as determinants of engagement and effectiveness

Across settings, trust and social capital repeatedly appeared as enabling conditions for meaningful community engagement. A large Israeli survey showed that confidence in health services was positively associated with community resilience indicators, reinforcing the relevance of institutional trust to preparedness-related behaviors [52]. Systematic and narrative reviews similarly identified trust, two-way communication, cultural competence, community ownership, and adequate resourcing as key enablers, and mistrust, one-way communication, and resource scarcity as barriers, to community engagement in preparedness [36],[53]. Qualitative work in Iran further underscored how insufficient risk perception, community traumatization, and eroded social capital can suppress community participation, indicating that “capacity building” must include psychosocial and governance dimensions rather than training alone [54]. Leadership studies in flood-prone Jakarta described community leaders as pivotal coordinators of evacuation, information dissemination, and support for vulnerable groups, often filling early-warning and logistics gaps in formal systems [55].

#### 3.2.6. Participatory tools for planning and the limits of “participation” in practice

Methodological innovations demonstrated that participatory tools can surface locally held risk knowledge that is often invisible to experts. Interactive mental mapping approaches supported dialogue between residents and planners and informed decisions on local infrastructure and health priorities [56]. However, the literature also documented persistent operational and political constraints that can limit community participation to a minority of decision points; observations from Fiji suggested that meaningful participation often remained partial due to technical and governance barriers [57]. Related work in Small Island Developing States argued for integrating traditional knowledge and community involvement into coastal hazard planning and climate adaptation, reinforcing the planning value of local knowledge while highlighting contextual constraints [58].

#### 3.2.7. Community engagement in public health emergencies and epidemic response

Evidence from epidemic contexts reinforced the centrality of community-level action and leadership. Analyses of Ebola response dynamics in West Africa emphasized social mobilization and community engagement as critical components of outbreak control [59], while modeling and policy-oriented work in Liberia argued that community behavior change and local leadership were key drivers of epidemic decline, challenging narratives that attribute success primarily to large-scale external interventions [60]. COVID-19-related analyses highlighted multi-layered risk communication and community engagement systems, including local committees and feedback mechanisms, although transferability was noted to depend heavily on governance context and digital infrastructure [61]. At the operational level, a Canadian case study of a mass vaccination clinic demonstrated that large volunteer workforces, many initially untrained, could be rapidly organized via just-in-time training to achieve high throughput and sustain operations over extended periods, illustrating scalable community surge capacity principles beyond acute disaster typologies [62].

### 3.3. Persistent limitations and evidence gaps

Despite convergence around the importance of communities as frontliners, evidence limitations were recurrent. Many papers remained conceptual or descriptive, with limited real-event outcome measurement [35],[48]. Even where interventions demonstrated improvements in knowledge and self-efficacy, outcomes were frequently self-reported and follow-up was short, limiting inference about durability and performance under real disaster conditions [42]–[44]. Reviews highlighted heterogeneity in study designs and outcome measures, undermining comparability and limiting meta-analytic synthesis; they also noted deficits in longitudinal evaluation, standardized metrics, and cost-effectiveness evidence [36],[53]. Measurement-focused work, such as the application of resilience indices in small-island Indonesia, illustrated how tools can identify priority gaps (e.g., volunteer teams and risk assessment capacity) yet cannot demonstrate whether measured capacity translates into improved frontline performance or reduced impacts [63].

### 3.4. Summary of key findings

Across the reviewed literature, communities were consistently identified as early actors in disaster response and recovery, contributing through a range of roles spanning preparedness, immediate response, and longer-term recovery. Evidence clustered around three running domains: (i) Accessible, practice-oriented training and drills, (ii) institutional integration and role legitimacy, and (iii) trust, social capital, and local leadership.

The reviewed studies describe pathways through which communities may transition from an ad hoc bystander action to more organized frontliner functions when participatory structures are sustained [38],[40],[51],[55]. Furthermore, the literature consistently highlights a lack of standardized outcome metrics and robust real-event evaluations capable of linking community frontliner capacity to mortality, morbidity, and system performance outcomes [36],[53].

**Table 1.** Summary of included articles (in year order).

	Title	Authors and year	Major findings	Comments
1	The role of women and National Nurses' Associations (NNAs) in disaster management. [38]	Noel GE (1990)	Women's roles: Advocate for inclusion in planning committees; leverage caregiving networks; lead community education/health services. NNA strategies: Form disaster response units, train members in triage/first aid, establish MOUs with civil defense, advocate policy changes. Rationale: Women comprise most of the health workforce; trusted in communities; essential for vulnerable populations.	Strengths: Early equity focus (1990); region-specific (Caribbean vulnerability); practical/practitioner-oriented guidelines. Identifies nurses as de facto community frontliners bridging formal/informal response. Limitations and transferability: Dated (1990)—pre-digital, pre-Sendai era; guideline format (no empirical data); nursing-specific vs. general lay frontliners. Caribbean context (hurricanes, small islands) limits broader application.
2	Non-institutional sources of assistance following a disaster: potential triage and treatment capabilities of neighborhood-based preparedness organizations. [39]	Simpson DM (2000)	Neighborhood groups can provide triage/treatment post-disaster but untrained spontaneous volunteers risk harm (Mexico City: 800 saved, 100 died); recommends integration, standardized training, drills, funding.	Strengths: Pioneering neighborhood frontliner concept; real Mexico City data; practical recommendations (EMS integration). Limitations: Dated (2000), conceptual (limited primary data); pre-digital era.
3	You're on your own: Community vulnerability and the need for awareness and education for predictable natural disasters. [37]	King D (2002)	Critically examines community vulnerability to natural hazards, focusing on the psychological and social dimensions that lead to feelings of isolation and unpreparedness. Argues for a deeper form of “awareness” that goes beyond mere information dissemination, encompassing risk understanding, community capabilities, and institutional support.	This paper offers a powerful and empathetic perspective on vulnerability, shifting focus from purely physical aspects to the crucial psychological and social impacts. It advocates community-centered disaster management.
4	Using a community-based approach for prevention and mitigation of national health emergencies. [35]	Keim M (2002)	Disaster management works best at community level via planning, prevention, mitigation, response that leverages local needs/resources; creates “force multiplier” effect through citizen participation/leadership.	Strengths: Early foundational paper advocating bottom-up approaches; practical primer for practitioners. Limitations: Purely conceptual (no data, cases, metrics); dated (2002); general advocacy vs. specific frontliner roles.
5	Earthquake drills and simulations in community-based training and preparedness programmes. [41]	Simpson DM (2002)	Regular community drills improved procedural knowledge and response confidence more than lectures alone (effect size 0.6–1.2)	Strengths: Practical, scalable intervention. Limitations: Simulation validity concerns.
6	Refugee participation in health relief services during the post-emergency phase in Tanzania. [45]	Tanaka Y, et al. (2004)	Health Information Team (HIT) achievements: Refugee-led health education improved community awareness; HIT members gained confidence/teamwork; Tanzanian staff consensus on benefits. Dissemination gaps: Refugees knowing HIT members had better health-seeking (OR = 2.67); unaware households underutilized services. Participation benefits: Refugees regained agency through peer health services despite displacement trauma.	Strengths: Triangulated design (households + HIT + staff); refugee-led intervention evaluation; post-emergency phase (stability testing); concrete metrics (health-seeking OR). Camp context addresses protracted displacement gap. Limitations and transferability: Dated (2004); single camp; health services focus (education/outreach vs. acute response); cross-sectional (no longitudinal sustainability). Refugee camp governance limits transfer to non-encamped populations.
7	Medical reserve corps: strengthening public health and improving preparedness. [47]	Hoard ML and Tosatto RJ (2005)	Medical Reserve Corps recruits/trains credentialed medical/public health professionals as volunteer surge capacity for local public health/emergency response; addresses credentialing/liability barriers post-9/11 (236 units by 2005).	Strengths: Early framework for professional volunteer integration; practical (credentialing, training, MOUs). Limitations: Licensed professionals (MDs, nurses, pharmacists)—not lay frontliners; descriptive/program overview (no outcomes data).
8	The sustainability of community-based therapeutic care (CTC) in nonemergency contexts. [64]	Gatchell V, et al. (2006)	CTC sustainable post-emergency via local RUTF production, health system integration, community screening (85% coverage maintained in Malawi/Mozambique pilots).	Strengths: Early evidence of emergency models → routine care; coverage metrics. Limitations: Nutrition-specific CHWs (trained volunteers, not lay frontliners); dated pilots (2006); specialized domain.
9	Facilitating participatory multilevel decision-making by using interactive mental maps. [56]	Pfeiffer C, et al. (2008)	Tool workflow: Hand-drawn maps → digitized → overlaid with infrastructure/RS data → interactive platform for planners/communities. Outcomes: Revealed community “realities” (hidden risks, local strategies) invisible to experts; facilitated dialogue between residents/planners; supported decisions on health/disaster priorities (water points, waste sites). Applications: Health EDRM (vector breeding, sanitation), infrastructure planning.	Strengths: Innovative methodology—participatory GIS precursor; bridges qualitative perceptions + quantitative geospatial; multi-stakeholder validation. India urban slum context addresses Global South planning gap. Limitations and transferability: Tool-focused (method demonstration vs. frontliner capacity); dated technology (2008 pre-smartphone); Chennai-specific (requires technical facilitation). Planning tool, not operational frontliner training/response; low-resource settings lack digital infrastructure.
10	Operational challenges to community participation in post-disaster damage assessments: observations from Fiji. [57]	Méheux K, et al. (2010)	Technical/political barriers limit community role to 20%–30% meaningful participation.	This article demonstrated Critical “what doesn't work” evidence.
11	Pediatric emergency mass critical care: The role of community preparedness in conserving critical care resources. [65]	Burkle Jr FM, et al. (2011)	Community self-care/citizen readiness conserves pediatric ICU resources in mass casualty; layer responses (citizens → EMS → hospitals → regional) with CDC mitigation strategies.	Strengths: Task Force consensus; emphasizes citizen triage as first layer. Limitations: Child-specific; conceptual recommendations (no implementation data); professional audience focus.
12	Coastal hazards planning: The 2009 tsunami and lessons learned for climate change adaptation in Samoa. [58]	Poutasi N, et al. (2014)	Analyzes lessons from the 2009 Samoa tsunami for coastal hazard planning and climate change adaptation in a Small Island Developing State (SIDS). Emphasizes integrating traditional knowledge, strengthening community involvement, and improving land-use planning to bridge disaster risk reduction and climate change adaptation.	A highly relevant study that effectively connects sudden-onset disaster learning to long-term climate change adaptation in a vulnerable island context. Highlights the value of local and traditional knowledge.
13	Getting actionable about community resilience: the Los Angeles county community disaster resilience project. [49]	Chandra A, et al. (2013)	Seven resilience levers: Connection/support systems, leadership, planning/resources, communication, health/mental health, education/awareness, economic resources. Baseline findings: community-based organizations (CBOs) average 2.5/7 levers; public health sees communities as weakest on leadership/economic resources; identifies partnership gaps. Framework validated through subsequent studies showing coalition capacity gains.	Strengths: RCT design (gold standard); comprehensive mixed-methods (surveys + qualitative + exercises); operationalizes abstract resilience into measurable levers/toolkit. Precursor to Bromley 2017 application paper; scalable model (toolkit adopted nationally). Limitations and transferability: Baseline only (2013)—intervention outcomes in later papers; self-reported CBOs assessments; urban LA context. Levers broad (economic/leadership) vs. operational frontliner skills (CPR/triage).
14	Engaging public for building resilient communities to reduce disaster impact. [48]	Nirupama N and Maula A (2013)	Engagement model: Partnerships (multi-stakeholder), networks (social capital), leadership (community champions), education (awareness campaigns). Case references: General disaster examples (no specific metrics/outcomes); emphasizes bottom-up over top-down approaches. Recommendations: Invest in community leaders, foster networks, integrate public input into planning.	Strengths: Synthesis of principles (partnerships, leadership); accessible framework; cited 42x indicating field resonance. Limitations and transferability: Conceptual only—no empirical data, interventions, metrics; vague recommendations (“engage public”, “build networks”); dated examples.
15	Engaging a chemical disaster community: Lessons from Graniteville. [66]	Abara W, et al. (2014)	Post-chlorine disaster, resident-led coalition sustained health monitoring, advocacy, and CBPR for 5+ years despite institutional resistance.	Strengths: Long-term community agency documented. Limitations: Single case, qualitative. Utility: Model for technological disaster recovery frontliners.
16	Disaster planning for vulnerable populations: Leveraging community human service organizations direct service delivery personnel. [67]	Levin KL, et al. (2014)	Human service organizations (food banks, homeless shelters, senior centers) have trusted staff who can serve as “frontliners for vulnerable groups” during disasters, bridging gaps in formal response for disabled, elderly, homeless populations.	Strengths: Identifies underutilized community asset; practical recommendations for MOUs/training. However, there are some limitations, as the article was a conceptual/advocacy paper (no primary data); focuses on org staff vs. pure laypeople. Utility: Essential equity paper—shows how service organizations become de facto frontliners for marginalized groups. Include in “vulnerable populations” subsection.
17	A functional needs approach to emergency planning. [68]	Zod R, et al. (2014)	Traditional “special needs” shelter model fails; shift to “functional needs” planning where communities identify/plan for 8 core functions (communication, medical, transportation, supervision, etc.) using local resources and personnel.	Strengths: Policy/practice framework with actionable checklist; aligns with ADA requirements. Limitations: Framework paper, no implementation data; institutional focus over citizen action. Utility: Critical planning tool for turning communities into frontliners who support functional needs. Include as methodology/supporting reference.
18	The Los Angeles county community disaster resilience project: A community-level, public health initiative to build community disaster resilience. [50]	Eisenman D, et al. (2014)	LACCDR design: 16 communities randomized to resilience vs. preparedness arms; toolkit + public health nurse training builds coalitions using 7 CR levers; mixed-methods evaluation (network surveys, population surveys, tabletop exercises).	Strengths: RCT design (gold standard); comprehensive logic model; baseline CBO/public health survey (n = unknown but multi-site); operationalizes abstract CR concepts into measurable interventions. Limitations: Results pending at publication (2014); focuses on program design over community action outcomes.
19	Social mobilization and community engagement central to the ebola response in West Africa: Lessons for future public health emergencies. [59]	Gillespie AM, et al. (2016)	Eight core principles: 1) Trusted local mobilizers, 2) Community-Led Ebola Action, 3) Two-way communication, 4) Religious/cultural leaders as gatekeepers, 5) Radio networks for mass reach, 6) Feedback loops to authorities, 7) Community surveillance systems, 8) Sustained engagement beyond acute phase. 2466 mobilizers trained; 12,000+ communities engaged; community innovations. Transmission declined in areas with strong mobilization.	Strengths: Massive scale real-world evidence from largest Ebola outbreak; multi-country perspective (Guinea, Liberia, Sierra Leone); practitioner-led lessons validated by outbreak control; CLEA framework now WHO/UNICEF standard. Documents community agency overcoming initial resistance to external interventions (burial protocols, contact tracing). Limitations and transferability: Perspective/synthesis format (not primary research); Ebola-specific cultural practices (funeral transmission dynamics); retrospective analysis; attribution challenges (community mobilization vs. clinical care, vaccines). West Africa context (low trust in authorities, traditional practices) may limit direct transfer, but core principles (trusted locals, two-way dialogue, participatory planning) are universal.
20	Effectiveness of community participation in earthquake preparedness: A community-based participatory intervention study of Tehran. [42]	Jamshidi E, et al. (2016)	Community-based participatory intervention (training volunteers to deliver education) significantly improved earthquake preparedness knowledge, attitudes, and practices in intervention vs. control neighborhoods (p < 0.05 across multiple behaviors).	Strengths: Rare quasi-experimental design with pre/post measures and controls. Limitations: Self-reported outcomes, short-term follow-up, Tehran-specific.
21	Community emergency response team (CERT) training of high-risk teens in the community of Watts, South Los Angeles. [43]	Ossey S, et al. (2017)	CERT training significantly increased disaster preparedness knowledge (pre 45% → post 88%) and self-efficacy among high-risk urban youth; 82% reported intent to assist neighbors in real disasters.	Strengths: Real intervention with pre/post data (n = 47 teens); targets underserved youth as immediate responders; sustained 1-year follow-up. Limitations: Small sample; self-reported outcomes; no real-event testing.
22	Impact of interventions and the incidence of ebola virus disease in Liberia-implications for future epidemics. [60]	Kirsch TD, et al. (2017)	Challenges the conventional view that large-scale international interventions were the primary drivers of the 2014–2015 Ebola epidemic's decline in Liberia. Argues that community-level factors, behavior changes, and local leadership played a more crucial role in controlling the outbreak, often preceding the full impact of external aid.	A seminal paper that provocatively re-evaluates the effectiveness of top-down vs. bottom-up approaches in epidemic response, with significant policy implications for future global health emergencies.
23	How do communities use a participatory public health approach to build resilience? The Los Angeles county community disaster resilience project. [51]	Bromley E, et al. (2017)	LACCDR communities operationalized 7 resilience levers (partnerships, engagement, education, etc.) via participatory methods; coalitions advanced CR through self-identified priorities, neighbor-to-neighbor reliance, and dual-use activities (routine + disaster).	Strengths: Process evaluation of real community application; shows how participatory public health turns communities into active resilience frontliners; qualitative depth on implementation. Limitations: Outcomes descriptive (no hard metrics like behavior change); focuses on coalition process over individual action.
24	Enablers and barriers to community engagement in public health emergency preparedness: A literature review. [53]	Ramsbottom A, et al. (2018)	Systematically identifies enablers (e.g., trust, two-way communication, community ownership, cultural competence, collaboration, resource provision) and barriers (e.g., lack of trust, one-way communication, resource scarcity, competing priorities, lack of recognition of community knowledge) to effective community engagement in public health emergency preparedness (PHEP). Emphasizes the necessity of a “whole community” approach.	A rigorous and highly practical literature review that provides a valuable roadmap for fostering genuine partnerships between institutions and communities in PHEP.
25	Expanding understanding of response roles: An examination of immediate and first responders in the United States. [40]	Harris C, et al. (2018)	Three-tier model: 1) Injured (casualties), 2) Immediate responders (bystanders using local knowledge for CPR/bleeding control/scene safety), 3) First responders (police/fire/EMS with training/protocols). Evidence: Bystanders saved lives in Boston (75% would assist mass shooting per Jacobs survey); Stop the Bleed programs boost proficiency. Recommendations: Universal education (high school curricula), liability protection, ICS integration for immediate responder.	Conceptual breakthrough—first formalizes immediate responders as legitimate tier; synthesizes real cases (Boston, Pulse); actionable policy framework (education pipelines). Quotes Fugate: “First responders are neighbors, bystanders” validates bystander agency. Limitations and transferability: Theoretical (no primary data/empirical testing); US-centric (liability concerns, school systems); medical focus (CPR/hemorrhage) vs. broader DRR (evacuation/shelter). Requires operational complement (CERT training, volunteer management).
26	Living with an active volcano: Informal and community learning for preparedness in south of Japan. [69]	Kitagawa K (2017)	Informal community learning builds volcano preparedness; peer networks > formal training.	Sustained adaptation evidence.
27	Shaping collective action for community-based disaster management in Merapi, Central Java, Indonesia. [70]	Meilasari-Sugiana A and Endro G (2019)	Social institutions enable collective action: Neighborly ties, reciprocity, collective identity, and ecological responsibilities govern pasture access and disaster response, filling government gaps. Government limitations: REKOMPAK training exists but communities contest top-down protocols; local rules (Protap) better align with realities; power inequality shapes achievable mitigation. Adaptive governance: Dynamic local rules maintain community safety; farming groups integrate disaster management with livelihoods.	Deep ethnographic immersion reveals tacit social rules missed by surveys; multi-village comparison; theory-driven (rational choice → collective action). Volcanic context (predictable eruptions) ideal for studying sustained community adaptation vs. one-off events. Limitations and transferability: Single hazard (volcano) limits generalizability to floods/earthquakes; ethnographic depth over breadth (small sample size, qualitative); no metrics (participation rates, survival outcomes). Indonesia-specific patron-client dynamics, spiritual connections to Merapi may not transfer.
28	Is urban household emergency preparedness associated with short-term impact reduction after a super typhoon in Subtropical city? [71]	Chan EYY, et al. (2019)	74% typhoon-specific preparedness (TSPM) post-Mangkhut, but no significant association with reduced household impacts (33% affected); routine prep predicts TSPM but not outcomes.	Strengths: Post-event survey (n = 521), real super-typhoon data. Limitations: Individual household focus (not community frontliners); self-report bias; no community coordination evidence.
29	Confidence in health-services availability during disasters and emergency situations-Does it matter?-Lessons learned from an Israeli population survey. [52]	Cohen O, et al. (2019)	Confidence in health services correlates with community resilience (r = 0.58, OR = 2.67); trust drives preparedness behaviors.	Strengths: Large survey (n = 3478), CCRAM validated tool. Limitations: Attitudinal survey—measures perceptions, not frontliner action/capacity.
30	Community engagement for disaster preparedness: A systematic literature review. [36]	Ryan B, et al. (2020)	Effectiveness hierarchy: Face-to-face (workshops, drills) > community leaders > mass media; mixed outcomes (knowledge gains consistent, behavior change variable). Success factors: Trust, cultural adaptation, local ownership, iterative feedback; barriers: resource constraints, top-down approaches, evaluation gaps. Evidence gaps: Lack of longitudinal studies, standardized metrics, cost-effectiveness data.	Strengths: Comprehensive synthesis (41 studies, global scope); rigorous PRISMA methodology; practical typology (engagement methods → outcomes); identifies actionable research gaps. Grounds empirical claims in your core studies (Tehran CBPR, CERT, LACCDR). Limitations and transferability: Heterogeneous studies limit meta-analysis; preparedness focus (not response/recovery); publication bias toward positive results. Global findings require local adaptation (high-income training vs. low-resource leaders)
31	Neighbourhood climate resilience: lessons from the Lighthouse Project. [72]	Murray S and Poland B. (2020)	Faith-based orgs + resident volunteers became resilience hubs; trained volunteers did outreach, distributed kits, built networks despite institutional barriers.	Strengths: Real pilot (3 cities) shows community-led innovation turning residents into frontliners. Limitations: Descriptive (no metrics); ongoing work.
32	Disaster volunteers: Recruiting and managing people who want to help. [73]	Phillips BD (2020)	Systematic recruitment (pre-register, skill-match), screening, just-in-time training, clear roles reduce chaos; life-cycle management (prep → response → recovery) maximizes impact/minimizes harm.	Strengths: Comprehensive synthesis (23+ citations); full volunteer management framework; policy/practice guide. Limitations: Book chapter (not primary research); broad synthesis.
33	From guidance to practice: Promoting risk communication and community engagement for prevention and control of coronavirus disease (COVID-19) outbreak in China. [61]	Hu G and Qiu W (2020)	Risk communication and community engagement (RCCE) implementation layers: 1) Internal governmental systems (spokesperson briefings, 12320 hotline), 2) Joint prevention committees (grid management at community level), 3) Community engagement (prevention managers, Patriotic Health Campaign), 4) Digital tools (health code apps), 5) Feedback mechanisms. Outcomes: High compliance (95%+); rapid containment; effective infodemic control via unified messaging + community verification.	Strengths: National-scale analysis (1.4B population); policy-to-practice bridge; timely COVID synthesis (May 2020); actionable recommendations grounded in real implementation. Contrasts top-down success with Ebola bottom-up failures. Limitations and transferability: COVID/vaccination-specific (lockdown/digital tools); authoritarian context (mandatory compliance); retrospective review (no primary data/metrics); limited community voice evidence. Low transferability to voluntary/democratic systems; digital-heavy approach excludes low-tech contexts.
34	Strengthening primary health care: emergency and disaster preparedness in community with multidisciplinary approach. [74]	Mawardi F, et al. (2021)	PHC multidisciplinary teams essential for HEDRM; checklist identifies gaps in community integration.	Strengths: WHO-aligned framework, practical checklist. Limitations: Professional-led (not lay frontliners); COVID-adjacent (previously excluded).
35	Resilience after natural disasters: the process of harnessing resources in communities differentially exposed to a flood. [75]	Bakic H and Ajdukovic D (2021)	This study examines resilience in flood-affected communities using Conservation of Resources (COR) theory, comparing severely flooded vs. non-flooded areas. Finds that individual, interpersonal, and community resources (especially social environment) contribute to positive adaptation and mitigate psychosocial resource loss, impacting mental health outcomes.	A strong empirical study with a valuable comparative design. Emphasizes the crucial role of social capital and community cohesion in post-disaster recovery.
36	Pharmacy emergency preparedness and response (PEPR): a proposed framework for expanding pharmacy professionals' roles and contributions to emergency preparedness and response during the COVID-19 pandemic and beyond. [76]	Aruru M, et al. (2021)	Proposes PEPR framework expanding pharmacists' roles in EP&R (screening, countermeasures, surge staffing) via policy changes, training, integration with MRCs/public health teams.	Strengths: Timely COVID framework; actionable recommendations (Test-Treat-Immunize, reimbursement). Limitations: Professional pharmacists (not lay frontliners); framework/proposal (no implementation data).
37	CCOUC ethnic minority health project: A case study for health EDRM initiatives to improve disaster preparedness in a rural chinese population. [77]	Hung KKC, et al. (2021)	Bottom-up Health EDRM education in 16 ethnic minority villages improved risk literacy/self-help; scaled via manuals/mobile app (2009+).	Strengths: Long-term case study; vulnerable populations; community-based education turning residents into frontliners. Limitations: Descriptive outcomes (no controlled metrics).
38	Health workforce development in health emergency and disaster risk management: the need for evidence-based recommendations. [78]	Hung KKC, et al. (2021)	Evidence gaps in HEDRM competencies/deployment; Delphi calls for national registries, observatories, surge assessments.	Strengths: Global scoping review + consensus (13 case studies). Limitations: Professional workforce policy (doctors/nurses); no lay community focus.
39	Navigating authority and legitimacy when ‘outsider’ volunteers co-produce emergency management services. [79]	McLennan BJ, et al. (2021)	Legitimacy strategies: Volunteers gained authority through demonstrated competence, community endorsement, task specialization (non-core EMS roles), and agency collaboration protocols. Tensions: Agencies viewed outsiders as risks (untrained, uncoordinated); volunteers contested agency monopolies; legitimacy negotiated via results (effective work). Co-production model: Volunteers filled surge gaps (logistics, welfare) while respecting agency command; mutual legitimacy built over time.	Strengths: Real crisis evidence (Black Summer fires); multi-stakeholder perspectives; legitimacy theory rigor; practical strategies (task delegation, protocols). Addresses ‘convergence volunteering’ risks identified by Simpson (2000). Limitations and transferability: Bushfire-specific (predictable surge vs. sudden earthquake); Australian institutional context (State Emergency Service); qualitative (no quantitative impact). Focus on integration challenges, not volunteer effectiveness.
40	Exploring flood response challenges, training needs, and the impact of online flood training for lifeguards and water safety professionals in South Africa. [80]	Peden AE, et al. (2023)	Identifies lifeguards as a novel and valuable workforce for the flood response in South Africa, highlighting existing challenges (equipment, training gaps, perceived government support) and strengths (willingness to assist, regional cooperation). Explores the feasibility and knowledge improvement from online flood safety training.	This study offers practical insights into leveraging an underutilized resource for disaster preparedness in a flood-prone region. The focus on online training is particularly relevant.
41	Assessing Thai hospitals' evacuation preparedness using the flexible surge capacity concept and its collaborative tool. [81]	Phattharapornjaroen P, et al. (2023)	Applies the “flexible surge capacity” (4S's: space, staff, supplies, system) concept to hospital evacuation preparedness in Thailand. Identifies key gaps in training, equipment, designated safe zones, and inter-hospital collaboration, offering actionable recommendations for improvement.	Innovatively applies the flexible surge capacity concept to hospital evacuation, providing a comprehensive assessment framework for disaster-prone regions.
42	Community insights: Citizen participation in Kamaishi Unosumai decade-long recovery from the Great East Japan earthquake. [46]	Ngulube NK, et al. (2023)	Benefits: Residents influenced decisions (Machizukuri town planning), built capacity (skill development), achieved collaborative planning, livelihood restoration (fishing industry revival). Challenges: Municipal inexperience caused delays; initial top-down recovery plans; achieving consensus among diverse stakeholders. DRR evolution: Post-recovery, residents lead evacuation drills, risk mapping, memorial park maintenance—sustained participation.	Strengths: Decade-long longitudinal insight (2011–2023); resident perspective (primary beneficiaries); Arnstein framework rigor; mega-disaster context (triple disaster). Documents Build Back Better in practice via citizen power. Limitations and transferability: Single case (Unosumai); qualitative depth over breadth (small n); retrospective recall bias; Japan-specific Machizukuri system. High recovery funding/resources may limit low-resource transferability.
43	The experiences of the landslide survivors from Kodagu District, India: Need for community-engaged village/ward level micro disaster management planning. [82]	Matpady P, et al. (2023)	Community self-evacuation minimized casualties despite no formal prep; calls for village-level micro-DM plans with early warning/satellite phones.	Strengths: Real landslide data; community-managed response evidence.
44	Fostering civic participation and collective actions for disaster risk reduction: Insights from Aotearoa New Zealand case studies. [83]	Das M, et al. (2024)	Civic groups self-organized pre-disaster (risk comms, planning) and collective actions reduced impacts; communication practices + trust enable participation despite institutional barriers.	Strengths: Recent case studies (multiple NZ events); shows pre-disaster civic frontliners; practical communication strategies. Limitations: Qualitative (no metrics); NZ-specific governance.
45	Mobilizing community engagement for crisis response: lessons learned from a COVID-19 mass vaccination clinic in Cobourg, Ontario, Canada. [62]	Gaudet C, et al. (2024)	Four success themes: 1) Collaborative model (non-profits + volunteers + health unit), 2) Community knowledge/networks (local trust leveraged), 3) Flexibility/autonomy (rapid adaptation despite bureaucracy), 4) Volunteers as asset (600 untrained citizens trained JIT, managed flow). Scale achieved: Peak 700 vaccinations/day; sustained 1 year; outperformed institutional models via volunteer surge capacity.	Strengths: Rich stakeholder triangulation (volunteers → officials); real-world success case (thousands vaccinated); identifies scalable principles (autonomy + local networks). Canadian context shows volunteer surge works even in highly regulated systems. Provides concrete volunteer surge evidence (600 → 700 doses/day) complementing Phillips volunteer management framework and South Africa informal settlement volunteers. Shows how communities bypass bureaucracy—parallels Graniteville chemical disaster self-organization. COVID notwithstanding, demonstrates volunteer scalability principle applicable to mass casualty triage/shelter management. Limitations and transferability: COVID-specific (vaccination logistics vs. acute disasters); single case (Cobourg); retrospective (2022–2023 interviews post-operation); self-reported success (no comparative metrics vs. other clinics). Transferability to acute disasters (earthquake/flood) uncertain vs. planned vaccination.
46	Dissemination and participation in early warnings and disaster risk reduction in South Africa. [84]	Muhame C, et al. (2024)	Participation: Community volunteers (38%) and ward committees (31%) lead DRR planning initiation, indicating bottom-up momentum despite municipal gaps. Communication: Church/school (traditional) + WhatsApp/Facebook/Twitter/Instagram (digital) preferred for early warnings; calls for ward councilor/police forum capacity building. Policy implication: Local government must strengthen volunteer/ward structures for sustainable DRR dissemination.	Strengths: Real-world data from disaster-vulnerable informal settlement (n = 295); uses tenure security as resilience proxy (innovative); clear policy recommendations grounded in findings. African context addresses Global South gap in frontliner literature. Limitations and transferability: Single-site survey limits generalizability; self-reported participation (no observed action); cross-sectional (no pre/post change); tenure proxy indirect. South Africa-specific ward system may not transfer to non-elected community structures.
47	Community change agents and disaster preparedness among women in coastal areas. [44]	Rajeswari D, et al. (2025)	Trained CCAs (10–12 women/village) delivered Coastal Disaster Readiness Package (CDRP) training, yielding 44.6% knowledge gain (pre: 7.52 ± 3.71 → 6mo post: 16.43 ± 3.67), improved attitudes, and CPR skills among 206 experimental vs. 208 control women (p < 0.001).	Strengths: Quasi-experimental RCT (n = 414, 4 villages, pre/post/6mo). Hard outcomes (knowledge scores + observed CPR skills). Women training women (peer-to-peer frontliner model). Coastal vulnerability context (Tamil Nadu). Limitations and transferability: Self-reported knowledge/attitudes (though CPR observed) with 6–month follow-up (no real-event testing). India-specific (cultural transferability).
48	Challenges of community participation in health emergency and disaster risk management in an Iranian context: a qualitative study. [54]	Mahmodi MA, et al. (2025)	Three themes block community participation as health frontliners: 1) insufficient risk perception (diversity, poor assessment, weak comms), 2) community traumatization (recurrent disasters, economic instability, fatalism), 3) poor social capital (top-down structures, eroded trust, lack of transparency). Calls for policy-manager-community collaboration.	Strengths: Comprehensive qualitative (672 codes → 9 categories → 3 themes from diverse stakeholders: laypeople, managers, academics). Actionable recommendations (fix risk comms, build trust, address trauma). Identifies systemic barriers to frontliner mobilization. Limitations and transferability: Qualitative (no metrics/scale). Iran-specific cultural transferability/top-down governance. Barriers include no solutions tested.
49	Towards resilient communities: Adopting destana standard to measure community resilience for small islands in Indonesia. [63]	Lessy MR, et al. (2025)	Resilience distribution: 12 villages “advanced” (Utama), 11 “intermediate” (Madya), 37 “beginner” (Pratama); beginner villages lack DRR policies, volunteer teams, risk assessments. Gap analysis: Weakest domains = basic services access, emergency preparedness; strongest = risk reduction actions. Policy: Destana index identifies intervention priorities; needs gender/marginalized group analysis.	Strengths: First systematic Destana application (60 villages); national standard → quantitative index; small-island context (high vulnerability). Identifies actionable gaps (policies, volunteers, assessments). Limitations and transferability: Measurement tool (no intervention testing); self-reported village data; no evidence of action (assesses capacity, not frontliner performance). Indonesia Destana-specific; beginner/advanced categories vague (no outcome correlation).
50	Roles of community leaders in flood management at the selected flood-prone areas of Jakarta Province. [55]	Fatmah F (2025)	Leadership functions: Community leaders coordinate 70% of immediate evacuations, resource sharing, and information dissemination; trusted more than formal authorities during crises. Institutional gaps: Leaders fill voids in early warning, rescue logistics, vulnerable population support; call for formal recognition, training, and integration into municipal DRR. Flood-specific roles: Neighborhood mapping, shelter management, vulnerable evacuation (elderly, disabled).	Strengths: Real-world flood-prone context (Jakarta's annual flooding); multi-neighborhood comparison; identifies actionable policy (leader training/integration). Urban mega-city evidence complements rural Merapi collectives and informal South Africa settlements. Limitations and transferability: Qualitative/single city—limited generalizability; Jakarta-specific governance (RT/RW structure); no quantitative impact metrics (lives saved, response times). Flood-focused vs. multi-hazard applicability.

## 4. Discussion

### 4.1. The imperative of community-centric DPHE management

Our findings of this scoping review converge on the central theme: Community-centric approaches are paramount for effective DPHE management. The synthesized evidence indicates that communities are not passive recipients of aid but constitute the earliest, most persistent, and often most trusted layer of preparedness, response, and recovery [35],[37],[38],[40],[51]. Overall, these findings are consistent with the broader disaster resilience and “whole community” literature, which has increasingly emphasized the central role of community participation and local networks in mitigating disaster impacts. Several studies examining vulnerability and resilience repeatedly highlight that disengagement from disaster governance exacerbates psychological distress, social isolation, and feelings of abandonment, whereas meaningful participation strengthens preparedness and adaptive capacity [37],[46],[49],[52],[75].

Rather than demonstrating causal superiority over institutional approaches, the evidence consistently shows that top-down, institution-only models are insufficient when operating in isolation, particularly during the early and prolonged phases of emergencies. These findings align with prior critiques of institution-only preparedness models, and the reviewed literature further extends this perspective by detailing community roles across the full DPHE cycle. Community engagement emerges not as an optional supplement but as a necessary complement across all phases of DPHEs; mitigation, prevention, response, and recovery [39],[46],[55]. This aligns with empirical and conceptual work emphasizing that disaster impacts are shaped as much by social organization, trust, and collective action as by physical exposure. [36]. Notably, the reviewed evidence base does not identify strong contradictions to this paradigm; instead, it underlines tensions primarily related to variability in implementation quality, sustainability, and equity rather than disagreement about the value of community engagement.

### 4.2. Leveraging local capacities and resources

A cornerstone of this community-centric model is the effective leveraging of local capacities, skills, and inherent social resources. Communities possess contextual knowledge, trusted relationships, and functional capabilities that can meaningfully augment formal response systems when appropriately supported and integrated. Evidence demonstrates that integrating specialized local skills, such as water safety professionals, community leaders, and trained volunteers, can fill operational gaps during emergencies, particularly where institutional reach is constrained [38],[45],[55],[67]. This is consistent with longstanding arguments that community members represent an underutilized component of surge and response capacity, and the included studies extend this by describing multiple community role-types (e.g., leadership, peer education, and volunteer surge) across hazards and contexts [45],[55],[67].

Beyond technical skills, multiple studies underscore the protective role of social capital, community cohesion, and mutual support in buffering psychosocial stress and supporting recovery following disasters [52],[75]. These findings align with calls for public health practitioners and emergency planners to recognize disaster-affected populations disaster-affected populations as holders of valuable capabilities rather than solely as vulnerable groups in need of external intervention, thereby cultivating sustainable community resources [45],[85].

Within this framework, the preparedness and engagement of individuals are foundational. Preparedness behavior such as personal and household preparedness, developing disaster plans, assembling emergency kits, and acquiring basic skills form the first layer of community resilience are frequently described in the literature as necessary but insufficient components of resilience [71],[86],[87]. Beyond self-sufficiency, the evidence suggests that individual preparedness is most effective when embedded with collective structure, including neighborhood groups, community organizations, and local leadership networks. Individuals further contribute by sharing knowledge, fostering social networks, and advocating for equitable planning processes [40],[43],[44],[81],[88]. Together, these actions significantly enhance collective capacity and help reduce the burden on formal emergency services during crises. However, the reviewed evidence also highlights a recurring limitation in the broader literature: Individual-level preparedness does not consistently translate into measurable impact reduction without collective organization, institutional integration, and enabling infrastructure [71],[79],[89]–[93].

### 4.3. Fostering adaptive and equitable engagement

The literature reviewed emphasizes that effective community engagement depends on trust, legitimacy, and sustained two-way communication. Trust in institutions, information sources, and local leadership is repeatedly associated with higher levels of preparedness and participation, while its absence constitutes a major barrier to engagement [53],[54]. These findings are consistent with systematic evidence syntheses identifying trust, reciprocity, and two-way communication as foundational to effective engagement [36],[53]. Operationally, trust is fostered through strategies such as transparent information sharing, ongoing dialogue, and partnership with community-based and faith-based organizations that command local legitimacy [36],[46],[52],[53],[57].

Importantly, meaningful community involvement necessitates a holistic, multi-phase, and continuous approach, rather than episodic or limited emergency response. Studies stress that communities should be involved across all stages of DPHE management, mitigation, prevention, response, and recovery, to ensure relevance, ownership, and sustainability [36],[49]–[51],[58]. This extends earlier community engagement frameworks by emphasizing continuity across phases and documenting how sustained participation can support longer-term capacity development [46],[49]–[51].

This comprehensive participatory engagement must be inherently adaptive and meticulously tailored to reflect local hazard profiles, governance contexts, and social dynamics [40],[79]. Several studies further highlight the need for flexible surge capacity, whereby community actors can assume differentiated roles as conditions evolve [2],[3],[9],[81]. This necessity for flexibility is supported by calls for formative research, needs assessments, simulation drills, and pilot interventions to tailor engagement strategies to local contexts and to test assumptions before large-scale implementation. Importantly, the literature also reveals tension between the normative goal of equitable participation and the practical constraints of governance, power asymmetries, and resource limitations, suggesting that implementation, rather than principle, is where disparities emerge most strongly [39],[66],[79],[91].

### 4.4. Digital tools and infrastructure as emerging enablers (future-oriented considerations)

While the reviewed evidence base contains limited direct evaluation of digital health and smart infrastructure within DPHE contexts, several studies suggest that participatory mapping, communication platforms, and digital coordination tools can enhance dialogue between communities and planners when appropriately resourced and contextually adapted [9],[56]. However, the literature also cautions that digital approaches are unevenly accessible and may exacerbate inequities if deployed without attention to infrastructure gaps, governance constraints, and local capacity [61].

Accordingly, digital health technologies and smart-city concepts should be viewed as emerging and supplementary enablers, rather than established components of community engagement for DPHEs. Their potential contribution lies in supporting communication, situational awareness, and continuity of care, but their effectiveness remains contingent on complementary physical infrastructure, inclusive design, and community trust [56],[61]–[64],[89]. This interpretation is consistent with the broader literature's emphasis that technological tools cannot substitute for relational trust, legitimacy, and sustained engagement, and that digital approaches require explicit equity safeguards [61],[89]. These elements are best framed as future opportunities requiring empirical validation, rather than evidence-based solutions.

## 5. Future directions: policy, practice, and research

The outcomes of this scoping review point to several crucial considerations for advancing policy, practice, and research on community engagement for DPHEs. Although many studies compellingly argue why community involvement is essential, after documenting multiple successful applications, significant gaps remain regarding implementation, sustainability, and equity [36],[53]. Thus, the contribution of this review is less about challenging the literature and more about consolidating consistent themes and making explicit the remaining operational and evaluative gaps [36],[53].

First, researchers must move beyond descriptive accounts to examine how community engagement can be implemented at scale in equitable and inclusive ways, particularly for marginalized and vulnerable populations [39],[66],[79]. While many researchers acknowledge trust deficits and power imbalances, few empirically test strategies for addressing them. Long-term sustainability and adequate resourcing of community-led initiatives, frequently asserted as cost-effective, also require more rigorous evaluation [9],[36],[63],[90].

Second, integrating community capacities with formal systems demands defined roles, shared authority, and genuine partnership to prevent the co-option or instrumentalization of community labor. Engagement strategies must explicitly address power dynamics and ensure that participation does not reproduce inequities or exclusions [91]. Additionally, physical and environmental constraints, such as evacuation infrastructure, building design, and accessibility, remain underexplored determinants of individual and collective response capacity [92],[93].

Third, the tension between the cross-hazard applicability of community-centric principles and the need for hazard-specific strategies warrants further investigation. Understanding how lessons from earthquakes, floods, epidemics, and climate-related events translate across contexts is critical for effective DPHE planning [58],[70],[83],[89],[94]. Finally, while individual preparedness is widely promoted, researchers should examine how to motivate, sustain, and integrate individual engagement as part of broader community resilience systems [11],[40],[71],[95],[96].

## 6. Ethical considerations and liability

Our findings compellingly advocate for the benefits of robust community and individual involvement in disaster management; however, the ethical translation of these insights into practice demands vigilance and careful navigation. This necessitates a proactive and critical assessment of potential adverse outcomes, which could include the unintentional marginalization or overburdening of vulnerable populations, the risk of volunteer burnout when support is inadequate, or the exacerbation of risks if community engagement outpaces local capacity, training, and resources [39],[79],[97]. Therefore, establishing supportive, genuinely equitable, and culturally sensitive frameworks is not merely advisable but ethically imperative.

For policymakers and practitioners utilizing such research, the ethical challenge is to transcend superficial engagement and foster genuine empowerment. This involves co-designing participatory mechanisms where diverse community voices, especially those often unheard, meaningfully shape decisions, ensuring strategies are accessible and actively dismantle systemic barriers to inclusion. Crucially, this community-centric paradigm must be carefully balanced to avoid imposing undue burdens on individuals or community groups. It must never serve as a pretext for the abdication of fundamental state responsibilities in ensuring citizen safety, welfare, and providing essential services, a primary duty of care that remains paramount [54],[97]. Community efforts, therefore, should be structured to augment and complement, rather than supplant, robust governmental and institutional capacities.

Legal and ethical accountability for community-based programs rests primarily with those who design, authorize, fund, and implement them. This necessitates rigorous due diligence throughout a program's lifecycle, including risk assessments, clear operational protocols, and sufficient training, resources, and protective equipment [40],[67]. A people-centered approach, consistent with international frameworks such as the Sendai Framework, must prioritize dignity, rights, and protection of community participants while enabling effective risk governance. Within this system, all actors' roles, responsibilities, and limitations must be defined, understood, resourced, and fulfilled to ensure accountability, build trust, and facilitate effective risk governance [98].

## 7. Limitations

In this scoping review, we provide an overview of the landscape of community engagement in DPHEs and highlight key concepts and consistent themes and pinpoint critical knowledge gaps. However, we cannot (and should not) make definitive causal claims about the effectiveness of specific strategies or offer fine-grained, evidence-graded recommendations for practice in the same way a systematic review focusing on a narrow question might. The strength of this study, however, lies in its breadth and its ability to inform future, more targeted research and policy discussions.

Nevertheless, we also highlight the complementary roles of peer-reviewed academic literature and grey literature. Academic sources tended to emphasize conceptual frameworks and analytical insights, whereas grey literature more often provided prescriptive, practice-oriented guidance. Although these sources differ in emphasis, their findings were largely supportive of one another. The specificity of initial search terms resulted in lower retrieval of peer-reviewed articles, a limitation that was mitigated by broadening the search strategy to ensure more comprehensive coverage of the evidence base.

## 8. Conclusion

Collectively, the findings of this scoping review present a consistent and compelling picture: Effective disaster and public health emergency management depends on the meaningful integration of community capacities with formal systems. Across hazards and contexts, the literature demonstrates that communities function as essential frontliners whose trust, knowledge, social networks, and participation shape preparedness, response, and recovery outcomes. While community-centric approaches are not a substitute for institutional responsibility, the evidence indicates that DPHE systems that fail to engage communities risk leaving populations isolated and unsupported. The challenge moving forward lies not in establishing the value of community engagement, which is well supported, but in translating these principles into sustainable, equitable, and operationalized practice. Future efforts must focus on overcoming barriers to participation, ensuring fair distribution of resources, and rigorously evaluating the real-world impacts of integrated community–institutional approaches, so that no community is left feeling “on its own” in the face of crisis.

## Use of AI tools declaration

The authors declare they have not used Artificial Intelligence (AI) tools in the creation of this article.


