# Supplementary material for: People as frontliners in the management of disasters and public health emergencies
Source: AIMS Public Health. 2026 Feb 27;13(1):240–72. doi: 10.3934/publichealth.2026014 (PMC13084393; doi:10.3934/publichealth.2026014)
Supplement: Supplementary file 1 [file publichealth-13-01-014-s001.pdf]

Public Health

https://www.aimspress.com/journal/aimsph

AIMS Public Health, 13(1): 240–272.
DOI: 10.3934/publichealth.2026014
Received: 17 September 2025
Revised: 09 January 2026
Accepted: 14 January 2026
Published: 27 February 2026

Review

People as frontliners in the management of disasters and public health emergencies

Phatthranit Phattharapornjaroen[1,2], Amir Khorram-Manesh[3,4,5], Gülcan Taşkıran Eskici[6], Yuwares Sittichanbuncha[2,7] and Lesley Gray[8,*]

[1] School of Paramedicine, Faculty of Health Science Technology, College of Medical Science, Chulabhorn Royal Academy, Bangkok 10210, Thailand
[2] HRH Princess Chulabhorn Disaster and Emergency Medicine Center, Chulabhorn Royal Academy, Bangkok 10210, Thailand
[3] Department of Surgery. Institute of Clinical Sciences, Sahlgrenska Academy, University of Gothenburg, Gothenburg 41345, Sweden
[4] Center for Disaster Medicine, University of Gothenburg, Gothenburg 40530, Sweden
[5] Gothenburg Emergency Medicine Research Group (GEMREG), Sahlgrenska University Hospital, Gothenburg 41345, Sweden
[6] Department of Nursing Administration, Faculty of Health Sciences, Ondokuz Mayis University, Samsun 57270, Türkiye
[7] Faculty of Health Science Technology, College of Medical Science, Chulabhorn Royal Academy, Bangkok 10210, Thailand
[8] Department of Primary Health Care, Faculty of Medicine, University of Otago, Wellington 6242, New Zealand

* **Correspondence:** Email: lesley.gray@otago.ac.nz; Tel: +6402102939729.

Table S1. Assessment using the Joanna Briggs Institute (JBI) critical appraisal checklist and Critical Appraisal Skills Programme (CASP) checklist.

	Title	Study design	JBI checklist type	JBI score (Yes/Total)	CASP result
1	The role of women and National Nurses' Associations (NNAs) in disaster management. [38]	Expert opinion	(***)	5/6	Moderate
2	Non-institutional sources of assistance following a disaster: potential triage and treatment capabilities of neighborhood-based preparedness organizations. [39]	Narrative	(*)	4/6	Moderate
3	You're on your own: Community vulnerability and the need for awareness and education for predictable natural disasters. [37]	Narrative	(*)	5/6	High
4	Using a community-based approach for prevention and mitigation of national health emergencies. [35]	Expert opinion	(***)	5/6	Moderate
5	Earthquake drills and simulations in community-based training and preparedness programmes. [41]	Quasi-Exp	(*****)	7/9	High
6	Refugee participation in health relief services during the post-emergency phase in Tanzania. [45]	Cross-Sectional	(**)	7/8	High
7	Medical reserve corps: strengthening public health and improving preparedness. [47]	Expert opinion	(***)	4/6	Moderate
8	The sustainability of community-based therapeutic care (CTC) in nonemergency contexts. [64]	Case report	(****)	6/9	Moderate
9	Facilitating participatory multilevel decision-making by using interactive mental maps. [56]	Qualitative	N/A	N/A	High
10	Operational challenges to community participation in post-disaster damage assessments: observations from Fiji. [57]	Case report	(****)	7/9	High
11	Pediatric emergency mass critical care: The role of community preparedness in conserving critical care resources. [65]	Expert opinion	(***)	6/6	High
12	Coastal hazards planning: The 2009 tsunami and lessons learned for climate change adaptation in Samoa. [58]	Case report	(****)	8/9	High
13	Getting actionable about community resilience: the Los Angeles county community disaster resilience project. [49]	Quasi-Exp	(*****)	9/9	High
14	Engaging public for building resilient communities to reduce disaster impact. [48]	Narrative	(*)	4/6	Moderate
15	Engaging a chemical disaster community: Lessons from Graniteville. [66]	Case report	(****)	8/9	High
16	Disaster planning for vulnerable populations: Leveraging community human service organizations direct service delivery personnel. [67]	Expert opinion	(***)	5/6	Moderate
17	A functional needs approach to emergency planning. [68]	Narrative	(*)	6/6	High
18	The Los Angeles county community disaster resilience project: A community-level, public health initiative to build community disaster resilience. [50]	Protocol	(*****)	8/9	High
19	Social mobilization and community engagement central to the ebola response in West Africa: Lessons for future public health emergencies. [59]	Narrative	(*)	6/6	High

Continued on next page

	Title	Study design	JBI checklist type	JBI score (Yes/Total)	CASP result
20	Effectiveness of community participation in earthquake preparedness: A community-based participatory intervention study of Tehran. [42]	Quasi-Exp	(*****)	8/9	High
21	Community emergency response team (CERT) training of high-risk teens in the community of Watts, South Los Angeles. [43]	Quasi-Exp	(*****)	7/9	High
22	Impact of interventions and the incidence of ebola virus disease in Liberia-implications for future epidemics. [60]	Narrative	(*)	6/6	High
23	How do communities use a participatory public health approach to build resilience? The Los Angeles county community disaster resilience project. [51]	Qualitative	N/A	N/A	High
24	Enablers and barriers to community engagement in public health emergency preparedness: A literature review. [53]	Syst. review	SR	10/11	High
25	Expanding understanding of response roles: An examination of immediate and first responders in the United States. [40]	Narrative	(*)	6/6	High
26	Living with an active volcano: Informal and community learning for preparedness in south of Japan. [69]	Case report	(****)	7/9	High
27	Shaping collective action for community-based disaster management in Merapi, Central Java, Indonesia. [70]	Case report	(****)	9/9	High
28	Is urban household emergency preparedness associated with short-term impact reduction after a super typhoon in Subtropical city? [71]	Cross-Sectional	(**)	6/8	Moderate
29	Confidence in health-services availability during disasters and emergency situations-Does it matter?-Lessons learned from an Israeli population survey. [52]	Cross-Sectional	(**)	7/8	High
30	Community engagement for disaster preparedness: A systematic literature review. [36]	Syst. review	SR	11/11	High
31	Neighbourhood climate resilience: lessons from the Lighthouse Project. [72]	Case report	(****)	7/9	High
32	Disaster volunteers: Recruiting and managing people who want to help. [73]	Expert opinion	(***)	5/6	Moderate
33	From guidance to practice: Promoting risk communication and community engagement for prevention and control of coronavirus disease (COVID-19) outbreak in China. [61]	Narrative	(*)	5/6	High
34	Strengthening primary health care: emergency and disaster preparedness in community with multidisciplinary approach. [74]	Narrative	(*)	4/6	Moderate
35	Resilience after natural disasters: the process of harnessing resources in communities differentially exposed to a flood. [75]	Cross-Sectional	(**)	8/8	High
36	Pharmacy emergency preparedness and response (PEPR): a proposed framework for expanding pharmacy professionals' roles and contributions to emergency preparedness and response during the COVID-19 pandemic and beyond. [76]	Expert opinion	(***)	5/6	Moderate

Continued on next page

	Title	Study design	JBI checklist type	JBI score (Yes/Total)	CASP result
37	CCOUC ethnic minority health project: A case study for health EDRM initiatives to improve disaster preparedness in a rural chinese population. [77]	Case report	(****)	7/9	High
38	Health workforce development in health emergency and disaster risk management: the need for evidence-based recommendations. [78]	Syst. review	SR	9/11	High
39	Navigating authority and legitimacy when 'outsider' volunteers co-produce emergency management services.	Qualitative	N/A	N/A	High
40	Exploring flood response challenges, training needs, and the impact of online flood training for lifeguards and water safety professionals in South Africa. [80]	Cross-Sectional	(**)	7/8	High
41	Assessing Thai hospitals' evacuation preparedness using the flexible surge capacity concept and its collaborative tool. [81]	Cross-Sectional	(**)	8/8	High
42	Community insights: Citizen participation in Kamaishi Unosumai decade-long recovery from the Great East Japan earthquake.	Case report	(****)	9/9	High
43	The experiences of the landslide survivors from Kodagu District, India: Need for community-engaged village/ward level micro disaster management planning.	Case report	(****)	8/9	High
44	Fostering civic participation and collective actions for disaster risk reduction: Insights from Aotearoa New Zealand case studies.	Case report	(****)	8/9	High
45	Mobilizing community engagement for crisis response: lessons learned from a COVID-19 mass vaccination clinic in Cobourg, Ontario, Canada.	Case report	(****)	9/9	High
46	Dissemination and participation in early warnings and disaster risk reduction in South Africa.	Cross-Sectional	(**)	7/8	High
47	Community change agents and disaster preparedness among women in coastal areas.	Quasi-Exp	(*****)	9/9	High
48	Challenges of community participation in health emergency and disaster risk management in an Iranian context: a qualitative study.	Qualitative	N/A	N/A	High
49	Towards resilient communities: Adopting destana standard to measure community resilience for small islands in Indonesia.	Cross-Sectional	(**)	6/8	Moderate
50	Roles of community leaders in flood management at the selected flood-prone areas of Jakarta Province.	Case report	(****)	8/9	High

Note: JBI = Joanna Briggs Institute (JBI) critical appraisal checklist. CASP = Critical Appraisal Skills Programme. *JBI Checklist for Textual Evidence Narrative, **JBI Checklist for analytic cross-sectional studies, ***JBI Checklist for expert opinion, ****JBI Checklist for case report, *****JBI Checklist for Quasi Experimental design, N/A = Not applicable.

AIMS Press

© 2026 the Author(s), licensee AIMS Press. This is an open access article distributed under the terms of the Creative Commons Attribution License (http://creativecommons.org/licenses/by/4.0)
